# Comparison of BNT162b2-, mRNA-1273- and Ad26.COV2.S-Elicited IgG and Neutralizing Titers against SARS-CoV-2 and Its Variants

**DOI:** 10.3390/vaccines10060858

**Published:** 2022-05-27

**Authors:** Nigam H. Padhiar, Jin-Biao Liu, Xu Wang, Xiao-Long Wang, Brittany H. Bodnar, Shazheb Khan, Peng Wang, Adil I. Khan, Jin-Jun Luo, Wen-Hui Hu, Wen-Zhe Ho

**Affiliations:** 1 Department of Pathology and Laboratory Medicine, Lewis Katz School of Medicine, Temple University, Philadelphia, PA 19140, USA; nigampadhiar@temple.edu (N.H.P.); ljb217@126.com (J.-B.L.); xuwang@temple.edu (X.W.); xiaolong.wang0002@temple.edu (X.-L.W.); brittany.bodnar@temple.edu (B.H.B.); shazheb.khan@temple.edu (S.K.); peng.wang@temple.edu (P.W.); adil.khan@tuhs.temple.edu (A.I.K.); whu@temple.edu (W.-H.H.); 2 Center for Substance Abuse Research, Lewis Katz School of Medicine, Temple University, Philadelphia, PA 19140, USA; 3 Center for Metabolic Disease Research, Lewis Katz School of Medicine, Temple University, Philadelphia, PA 19140, USA; 4 Department of Neurology, Lewis Katz School of Medicine, Temple University, Philadelphia, PA 19140, USA; jin.jun.luo@temple.edu; 5 Department of Microbiology, Immunology & Inflammation, Lewis Katz School of Medicine, Temple University, Philadelphia, PA 19140, USA

**Keywords:** SARS-CoV-2, BNT162b2, mRNA-1273, Ad26.COV2.S, vaccine, antibody, neutralization, variants

## Abstract

Because the vaccine-elicited antibody and neutralizing activity against spike protein of SARS-CoV-2 are associated with protection from COVID-19, it is important to determine the levels of specific IgG and neutralization titers against SARS-CoV-2 elicited by the vaccines. While three widely used vaccine brands (Pfizer-BNT162b2, Moderna-mRNA-1273 and Johnson-Ad26.COV2.S) are effective in preventing SARS-CoV-2 infection and alleviating COVID-19 illness, they have different efficacy against COVID-19. It is unclear whether the differences are due to varying ability of the vaccines to elicit a specific IgG antibody response and neutralization activity against spike protein of the virus. In this study, we compared the plasma IgG and neutralization titers against spike proteins of wild-type SARS-CoV-2 and eight variants in healthy subjects who received the mRNA-1273, BNT162b2 or Ad26.COV2.S vaccine. We demonstrated that subjects vaccinated with Ad26.COV2.S vaccine had significantly lower levels of IgG and neutralizing titers as compared to those who received the mRNA vaccines. While the linear regression analysis showed a positive correlation between IgG levels and neutralizing activities against SARS-CoV-2 WT and the variants, there was an overall reduction in neutralizing titers against the variants in subjects across the three groups. These findings suggest that people who received one dose of Ad26.COV2.S vaccine have a more limited IgG response and lower neutralization activity against SARS-CoV-2 WT and its variants than recipients of the mRNA vaccines. Thus, monitoring the plasma or serum levels of anti-SARS-CoV-2 spike IgG titer and neutralization activity is necessary for the selection of suitable vaccines, vaccine dosage and regimens.

## 1. Introduction

As of 25 April 2022, the total number of fully vaccinated individuals in the United States exceeds 219 million (66.1% of the total U.S. population). Out of fully vaccinated individuals in the United States, 57.4% (125,944,285) are vaccinated with the BNT162b2 vaccine (Pfizer/BioNTech), 34.8% (76,333,723) with the mRNA-1273 (Moderna) vaccine, and 7.7% (16,936,775) with the Ad26.COV2.S (Janssen) vaccine [1]. Clinical trials have demonstrated that while two doses of either the BNT162b2 or mRNA-1273 vaccine had >90% efficacy in preventing symptomatic SARS-CoV-2 infection and alleviating COVID-19 illness, one dose of the Ad26.COV2.S vaccine showed approximately 67% efficacy [2,3,4]. This efficacy difference between the mRNA vaccines and Ad26.COV2.S vaccine could be due to the varying ability of the vaccines to stimulate a specific IgG antibody response and neutralization activity against SARS-CoV-2. It has been reported that specific anti-spike IgG titers correlate well to the magnitude of the neutralization activities against SARS-CoV-2 and its variants in symptomatic infections and convalescent subjects [5,6,7]. In addition, several studies showed that mRNA vaccine-induced neutralizing antibody levels are highly predictive of immune protection from symptomatic SARS-CoV-2 infection [8,9,10,11,12]. We previously demonstrated that there is a positive correlation between plasma IgG levels and neutralization activities against wild-type (WT) SARS-CoV-2 and its variants (D614G, UK-B.1.1.7, UK-B.1.525, and SA-B.1.351) in healthy individuals vaccinated with either the BNT162b2 or mRNA-1273 vaccine [13].

Accumulating evidence has shown that vaccine-elicited antibodies are less potent in neutralizing SARS-CoV-2 variants when compared to WT. Bates et al. demonstrated that BNT162b2 vaccine-induced neutralization titers against B.1.1.7 and B.1.351 variants were lower than those to WT SARS-CoV-2 [14]. Choi et al. reported that individuals vaccinated with mRNA-1273 had significantly lower neutralization activities against B.1.351, B.1.351-v1, B.1.351-v2, B.1.351-v3, P.1, and B.1.617.2 variants compared to those to WT SARS-CoV-2 [15]. A study by Zhou et. al. showed that there was decreased neutralizing ability against the B.1.526 variant with the E484K mutation among BNT162b2 and mRNA-1273 vaccine recipients [16]. Moreover, mRNA vaccine-elicited antibody response varies among these individuals and reduced neutralizing activity against SARS-CoV-2 and the variants has been recognized after vaccination [17,18,19,20]. Therefore, these studies underscore the necessity to examine the differences in the antibody and neutralizing titers elicited by the vaccines against not only WT SARS-CoV-2 but also its variants. However, there is little information about comparing the specific IgG and neutralization titers against SARS-CoV-2 and its variants in healthy individuals vaccinated by the three commonly used vaccines in the United States. In this study, we examined the plasma IgG and neutralization titers against spike proteins of WT SARS-CoV-2 and its variants in healthy subjects who received either the mRNA-1273, BNT162b2 or Ad26.COV2.S vaccine.

## 2. Methods

### 2.1. Sample Collection

The Institutional Review Board of Temple University approved this research (IRB number: 28021). The inclusion and exclusion criteria for subject selection are shown in Table S1. Written informed consent was obtained from all subjects. A total of 84 subjects, of which 48 received two doses of BNT162b2, 26 received two doses of mRNA-1273 and 10 received one dose of Ad26.COV2.S, were recruited in the study. Blood was obtained between 22–138 days after the 2nd dose for BNT162b2 subjects (mean = 61.2 days), 24–182 days after the 2nd dose for mRNA-1273 subjects (mean = 58.2 days), and 42–239 days after the 1st dose for Ad26.COV2.S subjects (mean = 128.5 days). The age of study subjects ranged from 20 to 79 years for the BNT162b2 group (mean = 42.90), 20 to 79 years for the mRNA-1273 group (mean = 45.9 years) and 20 to 74 years for the AD26.COV2.S group (mean = 42.9 years). For each donor, at least 10 mL of whole blood was obtained, from which plasma was separated and used for the experiments.

### 2.2. Anti-Spike S1 IgG Quantification

The LEGEND MAX^TM^ SARS-CoV-2 SPIKE S1 Human IgG Elisa Kit from Biolegend (Catalog #: 447807) was used to quantify the anti-spike protein IgG antibodies in the plasma of study subjects. The analysis was performed as instructed by the manufacturer.

### 2.3. Recombinant VSV-Pseudovirus Synthesis

For the generation of the spike protein bearing VSV-based pseudoviruses, a pCAGGS vector was used to clone the spike protein sequence, with 18 codons at the C-terminal end of the sequence deleted to improve packaging efficiency. Mutations were induced using NEBuilder HiFi cloning, and sequencing was performed to ensure the required mutations. BHK-21/WI-2 cells (Kerafast, catalog #: EH1011) were transfected with the spike-expression plasmid and subsequently infected with rVSV-firefly-luciferase (Kerafast, catalog #: EH1020-PM) to yield VSV-pseudovirus containing a supernatant that was then collected, centrifuged at 500× *g* for 5 min, and then filtered with a 0.45 μm polyethersulfone membrane [21]. The variant mutation profiles were designed to match with the variants described by the WHO and online bioinformatics references [22,23].

### 2.4. Neutralization Assay

Hela cells bearing ACE-2 receptors (named Hela/Ace2-11 cells), gifted by Dr. Guangxiang Luo from the University of Alabama, were plated in 96-well tissue culture plates and cultured until reaching approximately 85% confluence. Donor plasma was diluted in 3-fold iterations (ranging from 1:16.67 to 1:36,450). Each dilution was incubated with spike protein-bearing pseudoviruses at 37 °C for 30 min. This plasma–pseudovirus mixture was then added to the cultured Hela-ACE2-11 cells for 36 h at 37 °C. The supernatant was then eluted and discarded, and the cells were washed with PBS before lysis with minimally auto-luminescent the lysis buffer from Promega (Catalog #: E1941, Madison, WI, USA). After waiting 8 min for cell lysis to occur, the plates were flash frozen in the −150 °C for 10 min to ensure 100% cell lysis. The cell lysis mixture was transferred to a Nunc^TM^ Microwell^TM^ 96-well Nunclon Delta-Treated White polystyrene plate (ThermoFisher Scientific, catalog #: 136101, Waltham, MA, USA). Firefly luciferase activity (relative luminescence unit; RLU) in lysates was measured using the luciferase assay system (Promega, catalog #: E1500) with EnVision Multimode Plate Reader (PerkinElmer, Waltham, MA, USA). The obtained RLU was normalized to those derived from cells infected with pseudovirus only. The half-maximal inhibitory dilution for plasma (ID_50_) was defined as 1/dilution and was determined using a four-parameter logistic curve (GraphPad Prism Version 9.1.1).

### 2.5. Statistics

A combination of GraphPad Prism (version 9.1.1) and Python (version 3.8.8) were used for statistical analysis. Luciferase data were normalized as % of positive control (which was pseudovirus with Hela/ACE-2-11 cells with no donor plasma), and then plotted as % of positive control vs. dilution. A four-parameter non-linear fit was applied for ID50 calculation. A Friedman’s one-way ANOVA was used to determine significant differences (*p* < 0.05) between WT and variants. The geometric mean (GM) of the ID_50_ for a given group (ex: mRNA-1273 IN-B.1.617.2) was calculated, and the geometric mean standard deviation factor (GSDF) was used to quantify the scatter of the data. The GSDF is a dimensionless and multiplicative measure of variation. The GM and GSDF of IgG titers for each group was also calculated. GM ID_50_ and IgG values from different vaccine groups were also plotted on the same graphs to allow for inter-vaccine comparisons. Significance was assessed with a Kruskal–Wallis one-way ANOVA with Dunn’s Multiple comparisons test (comparing each column to every other column). ID_50_ values and IgG titers were plotted on histograms to evaluate the distribution of the data, and bin widths were selected to avoid over-smoothing. Pearson’s correlation coefficients were calculated for the regression between plasma IgG values and ID_50_ values for each variant. *p* values less than 0.05 were statistically significant.

## 3. Results

### 3.1. Comparison of Vaccine-Elicited IgG and Neutralization Titers

**Figure 1:**
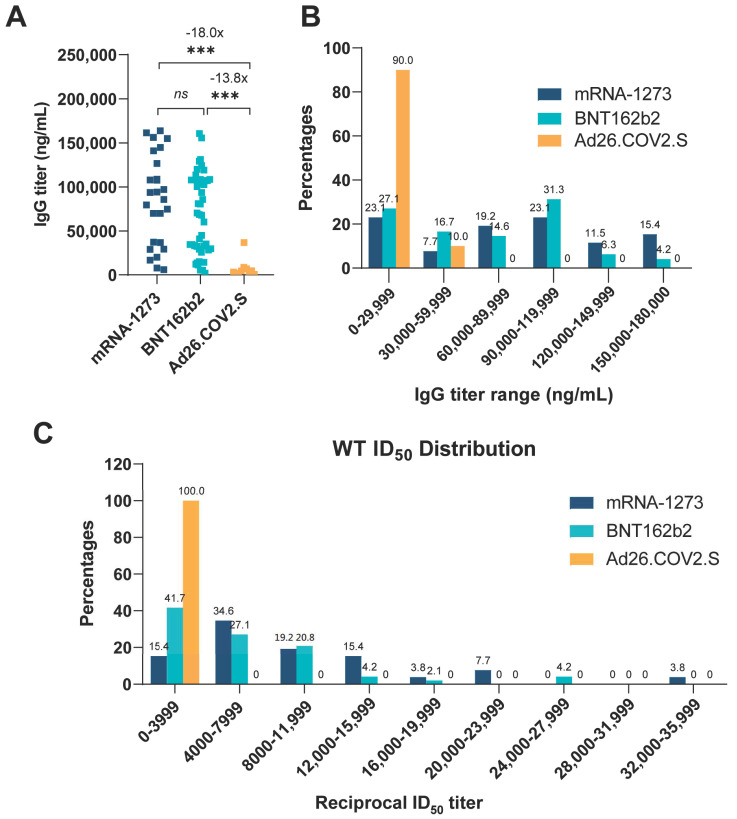
Distribution of specific anti-SARS-CoV-2 S1 IgG in plasma from vaccine recipients and distribution of ID_50_ values for SARS-CoV-2 WT. IgG titers and relative frequency for three vaccine groups (*n* = 10 for Ad26COV2.S, *n* = 26 for mRNA-1273 and *n* = 48 for BNT162b2). (**A**) Plasma anti-spike IgG titer comparison. Individual IgG titers in plasma collected from the vaccines are determined by ELISA assay and plotted for each vaccine group. A Kruskal–Wallis one-way ANOVA with Dunn’s Multiple comparisons test (comparing each column to every other column) was used to evaluate for statistical significance, which is defined as follows: *** (*p* ≤ 0.001). The numbers above asterisk indicate fold changes of IgG titers between two groups (the pairwise comparisons). (**B**) IgG titer frequency. Anti-spike IgG titers are depicted in 30,000 ng/mL intervals. The numbers on the top of each bar are the percentage of subjects whose IgG titers fall within the range shown on the abscissa axis. (**C**) Neutralizing ID_50_ values distribution of each vaccine group against SARS-CoV-2 WT. The numbers on the top of each bar are the percentage of subjects whose neutralizing ID_50_ titers fall within the range shown on the abscissa axis. GraphPad Prism version 9.1.1 was used for all statistical analysis for this figure.

We tested both plasma IgG antibody and neutralization titers against spike of WT SARS-CoV-2 and its eight variants (CA-B.1.429, CA-B.1.427, NY-B.1.526, NY-B.1.526-v1, NY-B.1.526-v2, IN-B.1.617.1, IN-B.1.617.2, and CO-B.1.621)**,** as demonstrated in Table 1. Subjects vaccinated with the mRNA-1273 had the higher mean of IgG titers (GM = 6.37 × 10^4^ ng/mL, GSDF = 2.53, 95% CI, 4.38 × 10^4^–9.27 × 10^4^ ng/mL) than those who received BNT162b2 (GM = 4.86 × 10^4^ ng/mL, GSDF = 2.75, 95% CI, 3.62 × 10^4^–6.53 × 10^4^ ng/mL), and the difference was not statistically significant (Figure 1A). However, when comparing the mRNA vaccine groups, the Ad26COV2.S group had significant lower mean of IgG titers, being 18-fold lower than the mRNA-1273 group and 13.8-fold lower than the BNT162b2 group (Figure 1A). The majority (90%) of subjects in the Ad26.COV2.S group had IgG titers in the lowest range (<30,000 ng/mL), and none of them had a titer higher than 40,000 ng/mL (Figure 1B).

**Figure 2:**
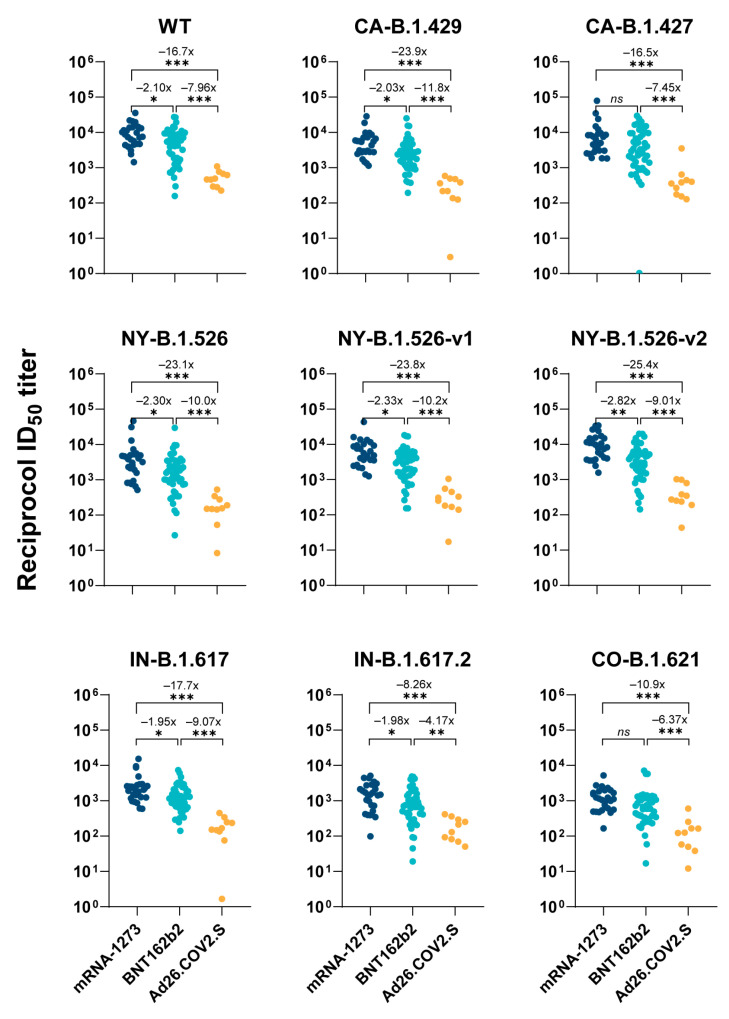
Inter-vaccine comparison of ID_50_ values for each variant. Plasma obtained from mRNA-1273-, BNT162b2- or Ad26.COV2.S-vaccinated subjects were collected. Neutralization was measured with recombinant vesicular stomatitis virus (rVSV)-based pseudovirus-bearing spike proteins of SARS-CoV-2 WT or the variants. The reciprocal neutralizing titers at a 50% inhibitory dilution (ID_50_, units = 1/dilution) against each SARS-CoV-2 variant are calculated for each donor and plotted in figure to compare among vaccine groups. A Kruskal–Wallis one-way ANOVA with Dunn’s Multiple comparisons test (comparing each column to every other column) was used to evaluate for statistical significance, which is defined as follows: *ns* (*p* > 0.05), * (*p* ≤ 0.05), ** (*p* ≤ 0.01), and *** (*p* ≤ 0.001). The numbers above asterisks indicate fold changes of geometric mean ID_50_ values between two groups (the pairwise comparisons). GraphPad Prism version 9.1.1 was used for all statistical analysis for this figure.

To examine pseudovirus-based neutralization activity against WT SARS-CoV-2 and the eight different variants, we used the GM for all ID_50_ values (50% half maximal inhibitory dilution, the dilution of plasma needed to neutralize 50% of positive control, units = 1/dilution) as well as the GSDF and 95% CI (Table 1). An inter-vaccine assessment of neutralizing titer (ID_50_) for all variants demonstrated the significantly lower titers (*p* < 0.01) in the Ad26.COV2.S group when compared to the mRNA groups (either mRNA-1273 or BNT162b2 group) (Figure 2). When comparing WT neutralizing activity, the mRNA-1273 (GM = 8.04 × 10^3^, 95% CI = 5.96 × 10^3^–1.08 × 10^4^, GSDF = 2.10) and BNT162b2 (GM = 3.83 × 10^3^, 95% CI = 2.73 × 10^3^–5.38 × 10^3^, GSDF = 3.21) groups had 16.7- and 7.95-fold higher ID_50_ (Figure 2) than the AD26.COV2.s group (GM = 482, 95% CI = 339–685, GSDF = 6.30).

**Figure 3:**
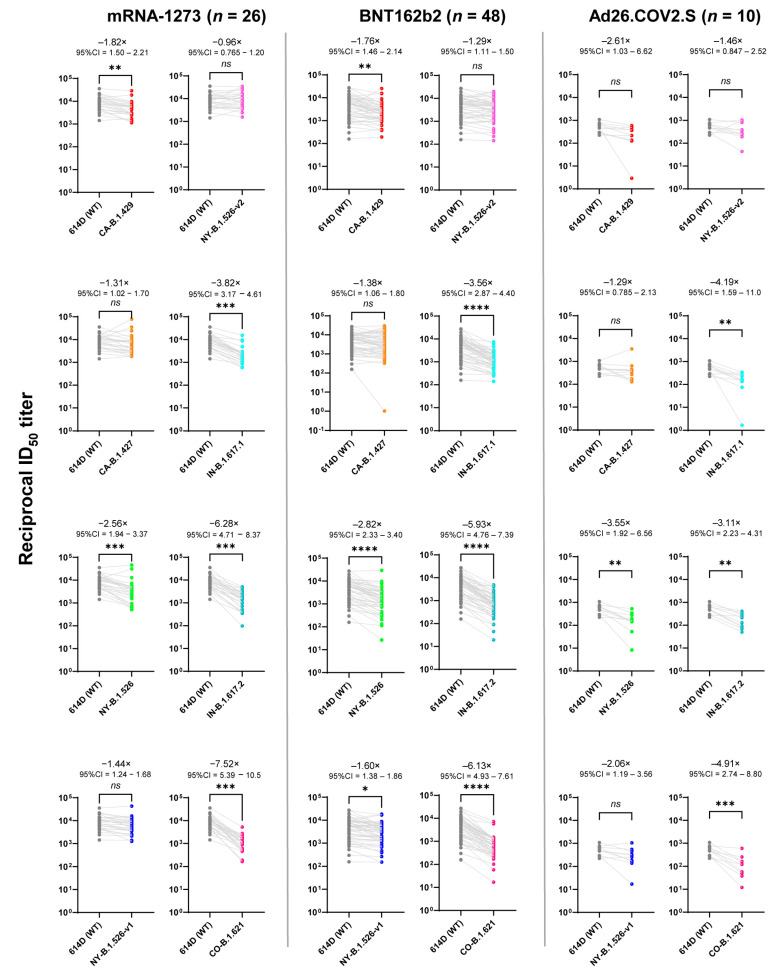
Neutralizing ID_50_ values for WT vs. variants in all three vaccine groups. Plasma obtained from mRNA-1273-, BNT162b2- or Ad26.COV2.S-vaccinated subjects were collected. Neutralization was measured with recombinant vesicular stomatitis virus (rVSV)-based pseudovirus-bearing spike proteins of SARS-CoV-2 WT or the variants. The reciprocal neutralizing titers at a 50% inhibitory dilution (ID_50_, units = 1/dilution) against each SARS-CoV-2 variant are calculated for each donor and plotted on figure to compare between SARS-CoV-2 WT and each variant. Friedman one-way ANOVA with Dunn’s multiple comparisons test (all variants compared to WT) was used to evaluate for statistical significance, which is defined as follows: *ns* (*p* > 0.05), * (*p* ≤ 0.05), ** (*p* ≤ 0.01), *** (*p* ≤ 0.001), and **** (*p* ≤ 0.0001). The fold changes in geometric mean ID_50_ values between two groups (the pairwise comparisons), as well as 95% CI for the fold change are shown above asterisk. The dots in figures indicate the ID_50_ titers of vaccinated individuals. GraphPad Prism version 9.1.1 in conjunction with Python version 3.8.8 was used for all statistical analysis for this figure.

While there was little difference in neutralization titers to the CA-B.1.427 and NY-B.1.526-v2 variants when compared to those of WT in the mRNA vaccine groups, there was an overall reduction in neutralizing titers against the variants in subjects across the three groups. As shown in Figure 3, when compared to WT SARAS CoV-2, the mRNA vaccine groups had significantly lower neutralization titers against the variants (CA-B.1.429, NY-B.1.526, IN-B.1.617.1, IN-B.1.617.2, and CO-B.1.621). For the mRNA-1273 group, a more than 6-fold decrease in neutralization titers was observed with the variants (IN-B.1.617.2 and CO-B.1.621) at −6.28-fold (95% CI = 4.71–8.37) and −7.52-fold (95% CI = 5.39–10.5), respectively. For the BNT162b2 group, the more than 5-fold reduction was observed with the IN-B.1.617.2 and CO-B.1.621 variants, and there were −5.93-fold (95% CI = 4.76–7.39) and −6.13-fold (95% CI = 4.93–7.61) decreases in neutralization titers, respectively (Figure 3). There were also diminished neutralization titers against the variants (NY-B.1.526, IN-B.1.617.1, IN-B.1.617.2, and CO-B.1.621) compared to WT SARS-CoV-2 in the Ad26.COV2.S with the lowest neutralization titers for the IN-B.1.617.1 variant (Figure 3).

### 3.2. Linear Regression Analysis

**Figure 4:**
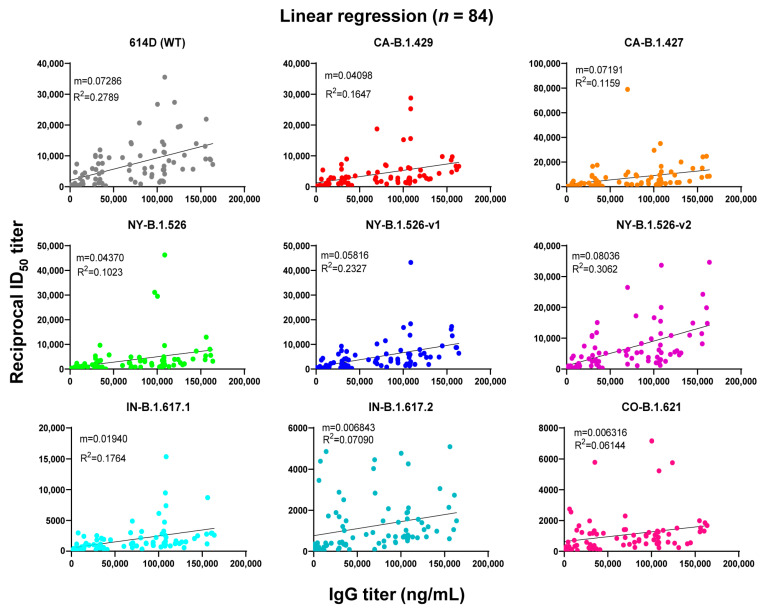
Linear regression for IgG titers vs. neutralizing ID_50_ values for WT SARS-CoV-2 and its variants. Shown is the correlation of the neutralizing titers ID_50_ (ordinate) and anti-SARS-CoV-2 spike S1 IgG levels (abscissa) of plasma from vaccinated subjects (*n* = 84 for vaccine group). Each figure contains the coefficient of determination (R^2^) as well as the slope (m). Significance was evaluated by calculating p values, which tests the null hypothesis that the slope is zero (no correlation). Linear regression analysis was performed using GraphPad Prism 9.1.1. software. Pearson’s correlation coefficients were calculated. Simple linear regression (solid line) is shown. R^2^ = goodness of fit. p-values less than 0.05 are statistically significant. All *p* values in each regression plot are <0.05.

As demonstrated in Figure 4, there was a positive correlation between IgG levels and neutralizing activities against SARS-CoV-2 WT and the variants, which was statistically significant (*p* < 0.01), with R^2^ values ranging from 0.0614 (CO-B.1.621) to 0.3062 (NY-B.1.526-v2)**.**

### 3.3. Supplementary Analyses

Figure S1 with the histograms of ID_50_ data shows that all variants of SARS-CoV-2 had leftwards shifts in their distribution, which points towards a lognormal pattern. Table S2 includes the mutation profile for each variant.

## 4. Discussion

In this study, we for the first time measured and compared the titers of the specific anti-spike IgG and neutralization titers of healthy vaccinees who received the different vaccines. When compared to the mRNA vaccine groups, the Ad26.COV2.S vaccine group had significantly lower plasma IgG levels and reduced neutralizing titers against WT SARS-CoV-2. This finding is supported by several clinical vaccine studies. Anand et al. examined the anti-RBD IgG response in dialysis patients vaccinated with different vaccines and showed that 83.3% of those who received Ad26.COV2.S vaccine did not develop RBD IgG antibody, compared with only 9.6% in the Pfizer-BioNTech vaccine group and 2.8% in the Moderna vaccine group [24]. In another study with a prospective cohort of organ transplant recipients, the authors demonstrated that anti-RBD antibody was detected in only 2 of 12 subjects who received Ad26.COV2.S vaccine compared to 59% of participants who received the mRNA vaccine series [25]. The same group also reported that patients with rheumatic and musculoskeletal disease had a lower rate of seroconversion following vaccination with Ad26.COV2.S compared to those who received the mRNA vaccines [26]. These findings in conjunction with ours indicate that the Ad26.COV2.S vaccine may elicit the limited humoral immune response against SARS-CoV-2. It is unclear whether the lack of the ability to elicit the stronger humoral immune response by the Ad26.COV2.S vaccine is due to use of a single dose as compared to the mRNA vaccines, which require two doses. When comparing the two mRNA vaccine groups, the mRNA-1273 group had higher IgG and neutralization titers against both WT and some of the variants, which is likely because the mRNA1273 vaccine contains a more than three-fold higher mRNA dose (100 μg) than that (30 μg) of the BNT162b2 vaccine. In addition, varying lipid nanoparticle composition in the two mRNA vaccines may also be a contributing factor to the discrepancy [27].

The neutralization titers against the SARAS-CoV-2 variants were generally lower than those to WT. The extent of decreased neutralization activities varied among the variants, which is associated with sites of the mutation. Jangra et al. reported that mutations in the receptor-binding domain (RBD) of the spike protein of SARS-CoV-2 may have more consequential impacts on neutralizing ability from vaccine-elicited antibodies [28]. There was decreased neutralizing ability against the B.1.526 variant with the E484K mutation among BNT162b2 and mRNA-1273 vaccinees [16]. Both IN-B.1.617.2 and IN-B.1.617.1 shared the distinct P681R mutation, which is a contributor to increased pathogenicity and fusogenicity [29]. In addition, the G142D mutation in these variants is implicated in enhancing viral transmission and immune evasion [30,31]. These studies underscore the necessity to determine the differences in the antibody and neutralizing titers elicited by the vaccines against not only WT SARAS CoV-2 but also its variants. We found that those variants with mutations at the 484 location (E484K for CO-B.1.621 and E484Q for IN-B.1.617.1) were more resistant to neutralization effects than other variants (Figure 3). We also observed that subjects of all three groups had a significant reduction in neutralization activities against both IN-B.1.617.2 (Delta) and IN-B.1.617.1 variants, particularly the Delta variant, which emerged as the dominant viral strain in late 2020 and was responsible for breakthrough infections (when compared to the pre-Delta era) [32,33]. In addition to the Delta variant, we showed that the CO-B.1.621 (Mu) variant was even more resistant to neutralization effects than the Delta variant (Figure 3). This observation agrees with the report that the Mu variant has been outcompeted by the Delta variant worldwide based on real-time tracking of mutant emergence through available bioinformatics resources [34].

While we showed that there is a positive and significant correlation between IgG titers and neutralization activities against WT SARS-CoV-2 and all variants, the correlations coefficient values are not particularly strong for some variants, with R2 values ranging from 0.06144 (variant CO-B.1.621) to 0.3062 (variant NY-B.1.526-v2) (Figure 4). The relatively low R values could be due to the small number of study subjects with a large range of age (18–80 years) and other unknown confounding factors. Studies have shown that vaccine-elicited antibody levels negatively correlated with age in study subjects who received the mRNA vaccines [35,36,37]. Because our study focused on the comparison of IgG and neutralizing titers in three different vaccine groups with age-matched subjects, examining the association between age and ability to produce antibodies is beyond the scope of this study.

## 5. Conclusions

In summary, our findings with vaccinated healthy subjects suggest that individuals who received one dose of the Ad26.COV2.S vaccine may have more limited IgG response and neutralization titers against SARS-CoV-2 and its variants than recipients of the mRNA vaccines. More clinical studies are needed to determine whether reduced IgG and neutralization titers of the Ad26.COV2.S vaccine is indeed associated with decreased protection from SARS-CoV-2 infection and development of severe COVID-19 disease. These future studies will further support the notion that monitoring the plasma or serum levels of anti-SARS-CoV-2 spike IgG titer and neutralization activity is necessary for the selection of suitable vaccines, vaccine dosage and regimens.

## 6. Limitations of the Study

There are several limitations of our work: First, because this study used in vitro pseudoviruses to test the neutralization activities against SARS-CoV-2 spike, we cannot conclude that anti-SARS-CoV-2 spike IgG and neutralization titers are associated with the clinical protection. Second, the impact of individual mutations (rather than group of mutations) of SARAS-CoV-2 spike on neutralization activity was not examined. Third, the Ad26.COV2.S group had a smaller number of subjects compared to the mRNA groups.

## Figures and Tables

**Table 1:** Descriptive statistics for each group, to include interquartile values, range, geometric standard deviation (SD) factor, and 95% confidence intervals (CI).

		WT	CA-	CA-	NY-	NY-	NY-	IN-	IN-	CO-	IgG
			B.1.429	B.1.427	B.1.526	B.1.526-v1	B.1.526-v2	B.1.617.1	B.1.617.2	B.1.621	Titers
**BNT162b2 Group (*n* = 48)**	**Minimum**	158	193	1	27	153	143	140	19	17	1960
	**Maximum**	27,400	25,300	29,500	29,500	18,400	20,000	7,390	4,860	7,160	160,000
	**Geometric mean**	3830	2170	2770	1360	2390	2970	1080	646	626	48,600
	**Geometric SD factor**	3	3	5	4	3	3	2	3	3	3
	**Lower 95% CI**	2730	1630	1720	939	1730	2130	838	462	456	36,200
	**Upper 95% CI**	5380	2900	4470	1970	3310	4150	1390	905	859	65,300
**mRNA-1273 Group (*n* = 26)**	**Minimum**	1440	1140	1830	514	1260	1580	594	98	165	6020
	**Maximum**	35,600	28,800	79,000	46,300	43,200	34,700	15,400	5100	5230	164,000
	**Geometric mean**	8040	4420	6120	3140	5570	8390	2100	1300	1010	63,700
	**Geometric SD factor**	2	2	3	3	2	2	2	3	2	3
	**Lower 95% CI**	5960	3220	4200	2010	4000	6020	1530	883	729	43,800
	**Upper 95% CI**	10,800	6050	8940	4910	7740	11,700	2900	1900	1410	92,700
**Ad26.COV2.S Group (*n* = 10)**	**Minimum**	224	3	129	8	17	44	2	50	12	746
	**Maximum**	1090	589	3520	530	1060	1030	340	413	601	36,900
	**Geometric mean**	482	185	372	136	234	330	115	155	98	3530
	**Geometric SD factor**	2	5	3	3	3	3	5	2	3	3
	**Lower 95% CI**	339	61	190	59	107	169	38	91	45	1520
	**Upper 95% CI**	685	560	730	311	514	645	350	265	215	8190

## Data Availability

The data presented in this study are available in the article and Supplementary Materials.

## Supplementary Materials

The following supporting information can be downloaded at: https://www.mdpi.com/article/10.3390/vaccines10060858/s1, Figure S1: Frequency distribution of ID_50_ titers for SARS-CoV-2 WT and 8 variants; Table S1: Inclusion and Exclusion Criteria; Table S2. Spike Protein Mutations in SARS-CoV-2 Variants.
