# Supplementary material for: Comparison of BNT162b2-, mRNA-1273- and Ad26.COV2.S-Elicited IgG and Neutralizing Titers against SARS-CoV-2 and Its Variants
Source: Vaccines (Basel). 2022 May 27;10(6):858. doi: 10.3390/vaccines10060858 (PMC9228110; doi:10.3390/vaccines10060858)
Supplement: Supplementary file 1 [file vaccines-10-00858-s001.zip › vaccines-1721900-supplementary.pdf]

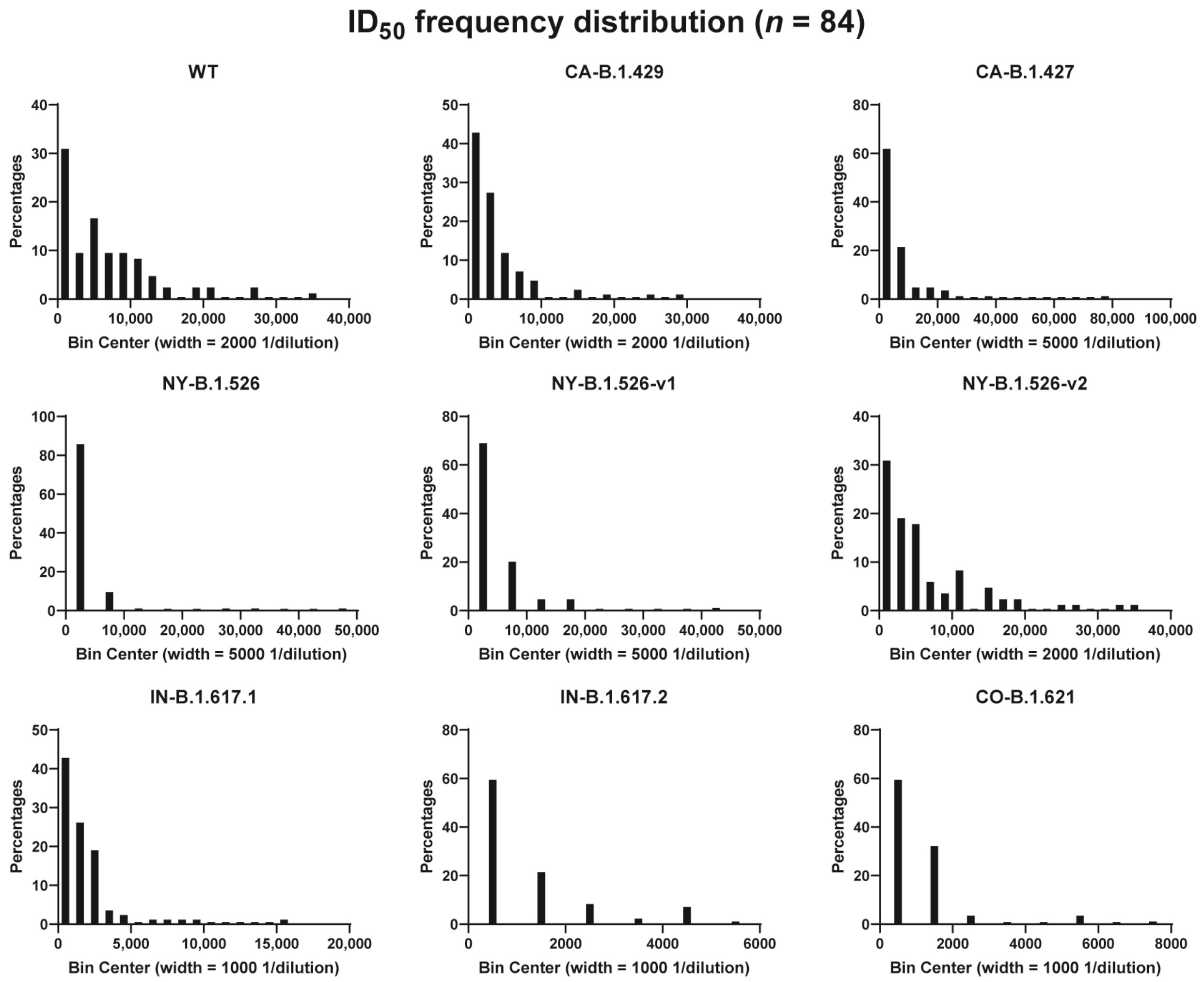

Figure S1. Frequency distribution of ID_{50} titers for SARS-CoV-2 WT and 8 variants. Bin width was selected for each group to allow for minimal over-smoothing of data.

Table S1. Inclusion and Exclusion Criteria.

**Inclusion Criteria**	**Subjects**
Age (years)	Age 18 – 80
Gender	Male and female
Race/Ethnic Group	Any race or ethnic group
Time point of blood collected	Three weeks to six months after the second dose of Pfizer or Moderna vaccine; six weeks to eight months after one dose of Janssen vaccine;
**Exclusion Criteria**	
	Not appropriately vaccinated
For female	Pregnant or lactating
Social history by Self-Report	No history of dependence or use of tobacco, alcohol and/or illicit drugs

General Health: by Self-Report	Severe chronic illness (cardiac, renal, pulmonary, endocrine, metabolic, diabetes or autoimmune disorders, and tuberculosis)
Viral infections	COVID-19/SARS-CoV-2, HIV, HBV, HCV
Others:	Abnormal liver function test, tuberculosis, feverish, dementia and cognitive decline

Table S2. Spike Protein Mutations in SARS-CoV-2 Variants.

SARS-CoV-2 Variant	Amino Acids Changes of Spike Protein
**CA-B.1.427 (Epsilon)**	L452R, D614G
**CA-B.1.429 (Epsilon)**	S13I, W152C, L452R, D614G
**NY-B.1.526 (Iota)**	L5F, T95I, D253G, E484K, D614G, A701V
**NY-B.1.526-v1 (Iota)**	L5F, T95I, D253G, S477N, D614G, A701V
**NY-B.1.526-v2 (Iota)**	L5F, T95I, D253G, S477N, D614G, Q957R
**IN-B.1.617.1 (Kappa)**	G142D, L452R, E484Q, D614G, P681R
**IN-B.1.617.2 (Delta)**	T19R, V70F, T95I, G142D, E156_F157del, R158G, A222V, W258L, K417N, L452R, T478K, D614G, P681R, D950N
**CO-B.1.621 (Mu)**	T95I, Y144S, Y145N, R346K, E484K, N501Y, D614G, P681H, D950N
